# Pregnancy-associated dense deposit disease with neurological complications caused by Salmonella Typhi: A case report

**DOI:** 10.5339/qmj.2026.45

**Published:** 2026-06-29

**Authors:** Sidra German, Daniya Tarique, Tajammul Waqar, Sajid Islam Bhatti, Huda Raja, Labiqa Khowaja

**Affiliations:** 1 Department of Nephrology, Sindh Institute of Urology and Transplantation (SIUT), Karachi, Pakistan

**Keywords:** Membranoproliferative glomerulonephritis, C3 glomerulopathy, dense deposit disease, typhoid fever, pregnancy complications, infection-related glomerulonephritis

## Abstract

**Background:**

Membranoproliferative glomerulonephritis (MPGN) is an uncommon cause of renal dysfunction and is increasingly being reclassified into immune complex-mediated and complement-mediated forms. Infections, particularly in endemic regions, may act as disease triggers. Pregnancy further complicates renal pathology through physiological and immunological changes.

**Case presentation:**

We describe a 22-year-old pregnant woman presenting with generalized edema, proteinuria, and cola-colored urine. Renal biopsy revealed diffuse endocapillary proliferation with mesangial and capillary wall C3 deposition, consistent with immune complex-mediated MPGN, with ultrastructural features suggestive of dense deposit disease (DDD). During hospitalization, she developed fever, papilledema, and neurological deficits. Blood cultures grew *Salmonella* Typhi, and urine cultures isolated vancomycin-resistant *Enterococcus*. Targeted antimicrobial therapy improved systemic symptoms, while cyclosporine was initiated for persistent proteinuria.

**Discussion:**

This case report illustrates an atypical presentation of DDD during pregnancy, potentially triggered by typhoid fever and complicated by neurological involvement. It underscores the diagnostic challenges in resource-limited settings and the complex interplay among infection, complement dysregulation, and gestation.

**Conclusion:**

This case report illustrates an atypical presentation of MPGN during pregnancy, triggered by typhoid fever and complicated by multidrug-resistant infection and neurological involvement. The patient’s renal and neurological symptoms improved with antibiotics and supportive care; however, the pregnancy resulted in intrauterine fetal demise at 28 weeks. Early recognition and multidisciplinary care were pivotal to maternal stabilization and fetal management.

## 1. INTRODUCTION

Chronic kidney disease (CKD) is a major global health problem, with glomerulonephritis (GN) being one of the leading causes of CKD.^1,2^ Membranoproliferative glomerulonephritis (MPGN) is a rare form of GN that has been reclassified into immune complex-mediated (IC-MPGN) and complement-mediated types (C3 glomerulopathy), reflecting advances in understanding its pathogenesis.^3^

Infection remains an important secondary trigger of MPGN, particularly in regions with high infectious disease burden such as South Asia.^4^ Pregnancy further complicates renal disease by increasing the risk of preeclampsia, maternal renal decline, and adverse fetal outcomes.^5^

Typhoid fever, caused by *Salmonella enterica* serovar Typhi, is endemic in South Asia and Africa and is associated with significant maternal–fetal morbidity, including sepsis, miscarriage, and preterm delivery.^6^ Although rare, renal involvement has been reported, ranging from proteinuria to acute GN.^7^

We report a rare case from Pakistan, managed at the Sindh Institute of Urology and Transplantation (SIUT), a tertiary-care referral center in Karachi. This case highlights the complex interplay of infection, complement dysregulation, and pregnancy in precipitating and complicating dense deposit disease (DDD).

Ethical approval for this case report was obtained from the SIUT review board. Written informed consent was obtained from the patient for publication of her clinical details and images.

## 2. CASE PRESENTATION

A 22-year-old pregnant woman from Dadu, Sindh, married for nine months, presented to the Emergency Department at the Sindh Institute of Urology and Transplantation with a 3- to 4-month history of progressive swelling and one month of joint pain. The swelling began in the feet and later involved the periorbital region. She reported mild, non-deforming polyarthralgia without morning stiffness, hair loss for 20 days, and two self-limiting episodes of cola-colored urine one month earlier. She denied oral ulcers, photosensitivity, rash, respiratory or cardiovascular complaints, neurological features, or gastrointestinal symptoms. Urine output was preserved, and her past medical and surgical history was unremarkable.

On admission in April 2024, she was conscious, oriented, and anemic, with bilateral pedal edema (2+). Blood pressure was 101/75 mmHg, and her pulse was regular at 90 beats per minute. Cardiovascular and abdominal examinations were unremarkable, while respiratory examination revealed basal crepitations. Ultrasound showed mildly echogenic kidneys with mild left-sided fullness and a viable intrauterine pregnancy of 17 weeks.

After initial stabilization with diuretics, a renal biopsy was performed in May 2024. Histopathology showed diffuse endocapillary proliferation with neutrophilic exudation and minimal tubular atrophy. Immunofluorescence revealed diffuse mesangial and capillary wall C3 deposits (++), with weak focal IgG and C1q, while IgA and IgM were negative. Electron microscopy (EM) demonstrated intramembranous dense deposits within the lamina densa, along with scattered mesangial and subendothelial deposits and diffuse podocyte foot process effacement, consistent with DDD. Anti-DNase B testing was unavailable in Pakistan, and C3 nephritic factor was negative.

She was observed for 24 h and discharged the following day in a stable condition on diuretics with outpatient follow-up. Two days after discharge, she developed severe lumbar pain and was readmitted on the fifth day after her initial discharge. Ultrasound excluded perinephric collection and renal vein thrombosis, while fetal ultrasound confirmed a viable 21-week pregnancy. During this admission, she developed fever, followed by headache, blurred vision, and numbness of both legs. Examination showed persistent anemia and edema, with a blood pressure of 105/63 mmHg. Neurological examination showed hyperreflexia with clonus and reduced motor strength (grade 3/5 on the MRC scale) in all limbs.

Despite a normal leukocyte count, empiric meropenem was started. Blood cultures grew *Salmonella* Typhi sensitive to second- and third-generation cephalosporins, and intravenous ceftriaxone 2 g daily was initiated. Urine culture revealed vancomycin-resistant *Enterococcus* sensitive to fosfomycin and linezolid. Cerebrospinal fluid (CSF) showed lymphocytic pleocytosis with sterile cultures and negative herpes simplex virus polymerase chain reaction (HSV PCR). Ophthalmology confirmed papilledema, while magnetic resonance imaging (MRI) of the brain and spine was unremarkable. Her neurological features improved with sensitivity-directed antibiotics, along with resolution of papilledema.

Oral cyclosporine was started at 3 mg/kg/day and titrated to maintain trough levels between 150 and 200 ng/mL, but was later discontinued due to elevated drug levels and lack of response. At 28 weeks of gestation, she delivered a stillborn fetus without postpartum complications. During six months of follow-up, she remained clinically stable with normal serum creatinine, improved serum albumin, and reduced proteinuria; however, the real-time PCR for *Salmonella* Typhi remained elevated.

## 3. DISCUSSION

This case represents an unusual presentation of DDD during pregnancy, further complicated by *Salmonella* Typhi bacteremia with neurological manifestations. Although MPGN and C3 glomerulopathies are recognized causes of nephrotic syndrome, DDD is rare, particularly in pregnant patients.^8^ Pregnancy induces significant alterations in renal physiology, complement regulation, and immune balance, which may exacerbate complement-mediated disease activity.

Reports of typhoid fever during pregnancy have primarily described maternal sepsis, miscarriage, and intrauterine fetal death, whereas renal complications are rarely documented.^9^ Even fewer cases have associated typhoid fever with immune-mediated glomerulopathies.^10^ To our knowledge, there are no previous reports of biopsy-proven DDD during pregnancy following *Salmonella* Typhi infection. This distinguishes our case by demonstrating how an infectious trigger in a susceptible host may accelerate complement dysregulation and promote dense deposit formation. Genetic testing for mutations in complement regulatory proteins (e.g., CFH, CFHR5) may help define the underlying etiology of C3 glomerulopathy; however, such testing was unavailable in our setting.^11^

The neurological manifestations in our patient add another layer of rarity. *Salmonella* Typhi has been reported to cause central nervous system complications such as encephalopathy and meningitis, although such findings are exceptional.^12^ In our case, papilledema and neurological deficits occurred in the context of typhoid bacteremia and improved following targeted antimicrobial therapy. This highlights the importance of considering extra-intestinal and extra-renal complications of typhoid fever in immunologically vulnerable patients.

Diagnostic challenges further distinguished this case. Genetic testing for complement regulatory protein mutations and Anti-DNase B titers, which are often useful in differentiating post-infectious GN, was unavailable in Pakistan. However, testing for C3 nephritic factor was negative, reducing the likelihood of persistent alternative pathway activation, as commonly seen in many cases of C3 glomerulopathy. Crucially, EM played a defining role in establishing the diagnosis by revealing dense intramembranous deposits characteristic of DDD, findings that would not have been identifiable with light microscopy and immunofluorescence alone. This underscores the indispensable role of EM in the diagnosis of C3 glomerulopathies.^13^

Our case aligns with prior literature suggesting that infections may act as triggers for complement activation and immune complex deposition leading to GN. However, it differs in two important aspects: (1) the coexistence of DDD with pregnancy, a scenario rarely documented, and (2) the concurrent neurological involvement due to *Salmonella* Typhi, which has not been previously reported in association with DDD.

Management was challenging due to the limitations of immunosuppressive therapy during pregnancy and the risks associated with uncontrolled infection. Supportive therapy, infection-directed antibiotics, and careful monitoring helped stabilize maternal renal function; however, the fetal outcome was unfavorable, a complication consistent with previous reports of adverse obstetric outcomes in women with significant renal disease.^14^

In summary, this case expands current knowledge by documenting pregnancy-associated DDD complicated by CNS infection with *Salmonella* Typhi. It underscores the importance of a multidisciplinary approach and maintaining vigilance for atypical presentations of glomerulopathies during pregnancy, especially in infection-endemic regions.

## 4. CONCLUSION

This case highlights a rare occurrence of pregnancy-associated DDD complicated by *Salmonella* Typhi-related neurological manifestations. Management required a multidisciplinary approach, including targeted antibiotic therapy for typhoid fever and cautious use of immunosuppression. Although the maternal renal and neurological status improved, the pregnancy resulted in intrauterine fetal demise at 28 weeks. The absence of genetic and Anti-DNase B testing, along with a negative C3 nephritic factor, posed diagnostic challenges; however, EM proved critical in confirming the diagnosis. Our report emphasizes the need for heightened clinical vigilance, multidisciplinary management, and access to advanced diagnostic tools in resource-limited settings, particularly when infections coexist with complement-mediated kidney disease during pregnancy.

## DATA AVAILABILITY

The data that support the findings of this study are available from the corresponding author upon reasonable request.

## ETHICAL APPROVAL

IRB approval was not taken, as according to our hospital guidelines, case reports are exempted.

## AUTHOR CONTRIBUTIONS

SG, DT, TG, HR, SIB and LK conceived the idea. SG and DT analyzed the data. TG, HR, SIB & LK wrote the manuscript, and proofreading was done by all authors.

## FUNDING

The author(s) received no financial support for the research, authorship, and/or publication of this article.

## ACKNOWLEDGEMENT

The author(s) have no acknowledgements to declare.

## DISCLOSURE OF AI USE

During the preparation of this work, the authors used DeepSeek for content assistance, specifically to summarize literature. The authors reviewed and edited the generated content as needed and take full responsibility for the final publication.

## CONFLICT OF INTEREST STATEMENT

The author(s) declare that there is no conflict of interest.

## References

[1] Levey AS, Coresh J (2012). Chronic kidney disease. Lancet.

[2] Lv J, Liu LJ, Zhang H (2025). Global burden of glomerulonephritis-related chronic kidney disease: 1990–2021. Int Urol Nephrol.

[3] Sethi S, Fervenza FC (2012). Membranoproliferative glomerulonephritis—A new look at an old entity. N Engl J Med.

[4] Nasr SH, Fidler ME, Valeri AM, Cornell LD, Sethi S, Zoller A (2011). Postinfectious glomerulonephritis in the elderly. J Am Soc Nephrol.

[5] Piccoli GB, Cabiddu G, Castellino S, Gernone G, Santoro D, Moroni G (2018). Kidney and pregnancy: Guidelines for nephrologists on the management of pregnancy in women with kidney disease. J Nephrol.

[6] Crump JA, Luby SP, Mintz ED (2004). The global burden of typhoid fever. https://iris.who.int/handle/10665/269150.

[7] Bhatt GC, Nandan D (2012). Salmonella typhi presenting as acute glomerulonephritis in twin siblings. Trop Doct.

[8] Fakhouri F, Fremeaux-Bacchi V (2022). Pathogenesis of C3 glomerulopathy. Front Immunol.

[9] Joshi S, Jaiswal S, Khan A (2020). Typhoid fever complicating pregnancy: A diagnostic and therapeutic challenge. J Family Med Prim Care.

[10] Masani N, Jhaveri KD, Fishbane S (2014). Update on membranoproliferative GN. Clin J Am Soc Nephrol.

[11] Zhang Y, Meyer NC, Wang K, Nishimura C, Frees K, Jones M (2012). Causes of alternative pathway dysregulation in dense deposit disease. Clin J Am Soc Nephrol.

[12] Huang DB, DuPont HL (2005). Problem pathogens: Extra-intestinal complications of Salmonella typhi infection. Lancet Infect Dis.

[13] Barbour TD, Pickering MC, Cook HT (2013). Dense deposit disease and C3 glomerulopathy. Semin Nephrol.

[14] Zhang JJ, Ma XX, Hao L, Liu LJ, Lv JC, Zhang H (2015). Outcomes of pregnancy in CKD: Systematic review and meta-analysis. Clin J Am Soc Nephrol.

